# Gravity, a regulation factor in the differentiation of rat bone marrow mesenchymal stem cells

**DOI:** 10.1186/1423-0127-16-87

**Published:** 2009-09-21

**Authors:** Yan Huang, Zhong-Quan Dai, Shu-Kuan Ling, Hong-Yu Zhang, Yu-Min Wan, Ying-Hui Li

**Affiliations:** 1 State Key Laboratory of Space medicine Fundamentation and Application, China Astronaut Research and Training Center, Beiqing Road, No.26, Beijing, China

## Abstract

**Background:**

Stem cell therapy has emerged as a potential therapeutic option for tissue engineering and regenerative medicine, but many issues remain to be resolved, such as the amount of seed cells, committed differentiation and the efficiency. Several previous studies have focused on the study of chemical inducement microenvironments. In the present study, we investigated the effects of gravity on the differentiation of bone marrow mesenchymal stem cells (BMSCs) into force-sensitive or force-insensitive cells.

**Methods and results:**

Rat BMSCs (rBMSCs) were cultured under hypergravity or simulated microgravity (SMG) conditions with or without inducement medium. The expression levels of the characteristic proteins were measured and analyzed using immunocytochemical, RT-PCR and Western-blot analyses. After treatment with 5-azacytidine and hypergravity, rBMSCs expressed more characteristic proteins of cardiomyocytes such as cTnT, GATA4 and β-MHC; however, fewer such proteins were seen with SMG. After treating rBMSCs with osteogenic inducer and hypergravity, there were marked increases in the expression levels of ColIA1, Cbfa1 and ALP. Reverse results were obtained with SMG. rBMSCs treated with adipogenic inducer and SMG expressed greater levels of PPARgamma. Greater levels of Cbfa1- or cTnT-positive cells were observed under hypergravity without inducer, as shown by FACS analysis. These results indicate that hypergravity induces differentiation of rBMSCs into force-sensitive cells (cardiomyocytes and osteoblasts), whereas SMG induces force-insensitive cells (adipocytes).

**Conclusion:**

Taken together, we conclude that gravity is an important factor affecting the differentiation of rBMSCs; this provides a new avenue for mechanistic studies of stem cell differentiation and a new approach to obtain more committed differentiated or undifferentiated cells.

## Background

The availability of sufficient, suitable cells is a limiting factor in regenerative medicine or cellular therapy which is a potential method for treatment of some diseases, such as myocardial infarction and bone defects. Mesenchymal stem cells (MSCs) are an attractive source of material for cellular replacement strategies for clinical applications and tissue engineering owing to their ability to replicate in the undifferentiated state, to differentiate into different cell lineages and the low immunogenic response *in vivo *[1-4]. Many studies have reported that MSCs can differentiate into cardiomyocytes after exposure to 5-azacydine (5-aza) [5,6], into osteoblasts in the presence of β-glycerophosphate and ascorbic acid-2-phosphate [7,8], and into adipocytes by treatment with dexamethasone [9]*in vitro *and *in vivo*. For instance, injection of MSCs pre-treated with 5-aza reduced the scar area of an infracted heart and improved damaged heart function in experimental animals [10-12]. The encouraging results of MSC differentiation studies in animal models led to clinical application studies, which indicated that MSCs may be an important and powerful cell source for regenerative tissue repair [13,14]. However, some problems remain to be resolved or improved, such as the amount of seed cells needed, committed differentiation and differentiation efficiency, before it can gain widespread application; committed differentiation and obtaining sufficient cells are particular problems and potential solutions have included exposing cells to hypoxia, specific growth factors, or low doses of chemical agents [15]. Most of these investigations have focused on the chemical microenvironment and the chemical signals that are thought to guide stem cells through the process of differentiation. Some researchers have tried to modify the MSCs by genetic engineering to express growth factors and signalling molecules, such as FGF2, SDF-1/CXCL12, angiopoietin, VEGF and Akt [15,16].

It is widely accepted that mechanical forces are important in the development, growth, the maintenance and function of tissues, such as the remodelling of skeletal muscle system and cardiovascular tissues. Recent evidence has shown that some mechanical factors such as fluid shear stress, mechanical strain and matrix rigidity can regulate the proliferation and differentiation of MSCs through various signalling pathways [17-20]. However, the effects of gravity on the differentiation of MSCs are not yet well understood.

Gravity is necessary to maintain the biological process from most tissues spreading all over the body. In the evolution of single-cell organisms to mammals, gravity has had an important role. In aquatic systems, gravity is balanced by a buoyancy force. However, when land-based organisms evolved, they had to counter 1 g gravity and developed perfect skeletal musculature and nervous control system to adapt to gravity conditions. When organisms are under microgravity condition, new changes happen. During the past 40 years of human spaceflight, it has been confirmed that exposure to microgravity affects almost all human physiological systems, with bone loss, anaemia, muscle atrophy and immune alterations commonly seen [21-24]. Previous research has shown that changes in the activity and differentiation of tissue cells are the main cause of physiological changes [25,26]. Our group and other researchers have demonstrated that simulated microgravity (SMG) inhibited the proliferation and osteogenesis of MSCs [27,28], and that gravity factors are involved in the differentiation of osteoblasts and skeletal muscle cells [29,30]. MSCs are important progenitor and supporting cells that have the intrinsic ability to self-renew and differentiate into multiple functional cells and are involved in the normal replacement of damaged cells and the disease-healing processes within different body systems, including force-sensitive (osteoblasts) and force-insensitive cells (adipocytes). There is increasing evidence that microgravity reduces cardiac contractility and bone strength, whereas hypergravity (HG) enhances the function of cardiomyocytes and bone [31,32].

To better understand if different gravity conditions affect the differentiation fate of MSCs and to identify a physical method to maintain MSCs in an undifferentiated state or promote MSCs committed differentiation, we investigated effects of different gravity conditions (SMG, HG, normal gravity) on the differentiation of MSCs into force-sensitive or force-insensitive cells and changes to the cytoskeleton and Erk1/2 phosphorylation levels, which are important in differentiation.

## Methods

### Isolation and culture of BMSCs

rBMSCs, rat BMSCs, were isolated and cultured *in vitro *using a previously reported, easy-handling method based on erythrocyte lysis developed by our laboratory [27]. In brief, 30-day-old male rats were sacrificed by cervical dislocation. Femora and tibia were isolated and sterilized with 0.75 volume fractions of ethanol for 5 min. After three rinses with 0.01 M phophate-buffered saline (PBS), each end of each femur and tibia were removed and douched with LG-DMEM medium. The medium was supplemented with 10% fetal calf serum (FCS; PAA, Austria), 100 U/ml penicillin, 100 μg/ml streptomycin, and 25 mM Hepes (all from Sigma, USA). The flushed mixture was filtered through a 400-screen mesh, then centrifuged for 10 min at about 300 g. The collected cells were re-suspended in medium and a 4-fold volume of red blood cell lysis buffer (0.15 M NH_4_Cl, 10 mM KHCO_3_, and 10 μM EDTA) was added. After 7-min incubation, the cells underwent centrifugation for 5 min at 1100 g resuspended in medium and then centrifuged twice more (5 min at 300 g); cells were resuspended in medium after each centrifugation procedure. The re-suspended cells were seeded to 25 cm^2 ^flasks at a density of with 2-3 × 10^5 ^cells per flask. When clones had formed at approximately 2-3 weeks later, the cells were digested with 0.25% trypsin and passaged to new flasks. After the first passage, the medium was changed every 3 days and passage was conducted when cells reached confluence. rBMSCs of passages 2-4 were used for the following experiments.

The primary cardiomyocytes were isolated from new born rats as previously described [33] and the ROS17/2.8 cells, an osteoblast cell line, were cultured in LG-DMEM medium. Both cells were used as positive controls for RT-PCR analyses.

### Cell culture and differentiation under HG/SMG

For the HG/SMG experiments, the cells were plated onto glass coverslips in six-well plates or into 25-cm^2 ^culture flasks. After the cells had adhered to the coverslips, at about 24 h later, the coverslips were transferred to a biocompatible polyethylene culture bag and stabilized with two bars at the edge of coverslip [34]. The samples (coverslips and flasks) were incubated with medium with or without chemical inducer, sealed ensuring that no air bubbles were present, then cultured on a cell centrifuger (developed by our laboratory) to obtain 2 G hypergravity, or a clinostat (MG3, developed by the Chinese Academy of Science Biophysics Institute) to simulate microgravity at a speed of 30 r/min [27]. The medium was changed every 3 days during HG/SMG culture if needed. For the cardiomyogenic differentiation experiments, the cells were incubated in normal medium without inducer under normal gravity condition and cultured to 21^st ^day from seeding after centrifugation or clinorotation culture for 1 or 3 days; during this period, cells were changed medium without inducer every 3 days. The chemical inducer used for cardiomyocyte differentiation was 5-aza (Sigma, USA) at a final concentration of 50 μmol/l. For osteogenic and adipogenic differentiation experiments, the cells were incubated in normal medium without inducer and cultured for 14 days after seeding after undergoing HG/SMG culture for 1, 3, 5 or 7 days. The chemical inducer used for osteogenesis was a mixture of 10 mM β-glycerophosphate, 50 μM ascorbic acid, and 100 nM dexamethasone. The adipogenic inducer used was 1 μM dexamethasone, 10 μg/ml insulin, 500 μM 3-isobutyl-1-methyl- xanthine, and 200 μM indomethacin. The control cells with or without inducer were also cultured for 21 days for cardiomyocyte or 14 days for osteoblast and adipocyte at normal gravity in the same incubator of HG group or SMG group respectively. At the end of the experiment time, the cells on coverslips were fixed with 4% paraformaldehyde and analyzed using the proliferation assay with the methylene blue method or the immunocytochemistry test. Some samples were treated with Trizol (Invitrogen, USA) for RNA extraction analysis; some samples were treated with lysis buffer (50 mM Tris pH 8.0, 150 mM NaCl, 0.02% NaN_3_, 0.1% SDS, 1% NP-40, 0.5% sodium deoxycholate, 2 mM PMSF, protease and phosphatase inhibitor cocktails; all from Sigma, USA) for analysis of total protein extracts; and some samples that were incubated without inducer were detached by 0.25% trypsin (Sigma, USA) for FACS analysis.

### Methylene blue method

Relative population cell density was measured using the methylene blue method as previously described [27]. Fixed cells were rinsed twice with PBS, stained with 1% methylene blue in borate buffer (10 mM, pH 8.8) for 10 min and then washed several times with borate buffer. Bound methylene blue was eluted with 0.1 mm/l HCl, and measurements were performed using a microplate reader (μQuant, Bio-Tech, Winooski, VT, USA) at 650 nm. Relative cell numbers are expressed as the absorption, OD, values.

### Immunocytochemistry

The cells for cardiomyocyte detection were fixed with 4% paraformaldehyde, then permeabilized with 0.1% Triton X-100 in PBS and blocked in 1% bovine serum albumin (BSA). The primary antibodies used were monoclonal antibodies to cTnT (Santa Cruz, USA), at a dilution of 1:100; cells were incubated with the antibodies for 120 min at room temperature (RT). After washing with PBS, horseradish-peroxidase (HRP)-conjugated antibodies (zhongshan, China) were added to the cells for 90 min at a dilution of 1:100. The signals were visualized using diaminobenzidine substrate and counterstaining using hematoxylin (Sigma, USA). The fixed cells for adipocyte detection were washed with PBS and stained with oil-red O (Sigma, USA) for 10 minutes, and then counterstained with hematoxylin for 3 minutes. Finally, the cells were observed and photographed using a DMLB microscope (Leica, German).

### RT-PCR analysis

Total RNA was extracted from cultured cells using Trizol reagent according to the manufacturer's protocol. Primers (synthesized by Sbsbio, China) for GATA4, β-MHC, ColIA1, Cbfa1, ALP, PPARγ2 and GAPDH are shown in Table 1. RT-PCR using Access RT-PCR Reagent (Promega, USA) was performed for 35 cycles; each cycle consisted of 94°C for 30 s, 55°C for 30 sec and 68°C for 1 min. Samples (10 μl) of each PCR product were size-fractionated by 1.5% agarose gel electrophoresis and the bands were visualized with ImageMaster VDS (Pharmacia Biotech, USA).

**Table 1:** Rat primers used for RT-PCR analysis.

Gene		Primer sequence	Length (bp)
GATA4	Forward	5'CTGTCATCTCACTATGGGCA	310 bp
	Reverse	5'CCAAGTCCGAGCAGGAATTT	
β-MHC	Forward	5'TGGCACCGTGGACTACAATA	100 bp
	Reverse	5'TACAGGTGCATCAGCTCCAG	
ColIA1	Forward	5'GTGGATGGCTGCACGAGTC	243 bp
	Reverse	5'TGAGTTTGGGTTGTTGGTCTGT	
Cbfa1	Forward	5'CACGACAACCGCACCATG	165 bp
	Reverse	5'GTCCCATCTGGTACCTCTCCG	
ALP	Forward	5'GGAAGGGTCAGTCAGGTT	366 bp
	Reverse	5'GTGGGCCGCTCTAGGCACCAA	
PPARγ2	Forward	5' TTGATTTCTCCAGCATTTC	360 bp
	Reverse	5' GCTCTACTTTGATCGCACT	
GAPDH	Forward	5'ACCACAGTCCATGCCATCAC	452 bp
	Reverse	5'TCCACCACCCTGTTGCTGTA.	

### Western-blot analysis

Total proteins of rBMSCs were extracted using a standard method and quantified using the BCA protein assay kit (Pierce, USA). Whole cell protein extracts (20 μg/lane) were separated by SDS-PAGE and transferred to a polyvinylidene difluoride (PVDF) Immobilon-P membrane (Millipore, USA) using a semi-dry electroblotter (CLP, USA). Protein transfer efficiency and size determination were verified using prestained protein markers (Sigma, USA). Membranes were blocked with 5% nonfat milk for 0.5 h at RT, followed by overnight incubation at 4°C with primary antibodies against cTnT, cbfa1 and phosphorylated ERK (all from Santa Cruz Biotechnology, USA) at dilutions of 1:1000. Primary antibody binding was detected using an HRP-conjugated secondary antibody and an enhanced chemiluminescence detection system (Amersham Bioscience, Piscataway, NJ).

### Flow cytometric assay

rBMSCs were trypsinized (0.125% trypsin) and washed with PBS twice. The supernatant was discarded and cells were incubated in 100 μl PBS and then blocked with 0.1% BSA for 10 min. The cells were stained according to the manufacturer's recommendations with monoclonal antibodies against cTnT or cbfa1 (Santa Cruz, USA) at RT for 60 min, then incubated with fluorescein isothiocyanate (FITC)-conjugated secondary antibodies at RT for 30 min. After incubation, cells were washed with PBS containing 0.1% BSA and resuspended in 0.5 ml PBS. Quantitative analyses were performed using a Beckman Coulter flow cytometer (Beckman Coulter, USA).

### Fluorescent staining of cytoskeleton

BMSCs were cultured under HG/SMG for 7 days. Cells were then fixed with 4% paraformaldehyde and permeabilized with 0.1% Triton X-100 for 10 min and blocked with 1% BSA (Sigma, USA). Primary antibodies, monoclonal antibodies to microtubules (Probe, USA), at a dilution of 1:100, were added to the cells for 120 min at RT. After washing with PBS three times, cells were incubated with FITC-conjugated antibody (Santa Cruz, USA) for 120 min at a dilution of 1:100. The cells were washed with PBS and incubated with Texas red isothiocyanate-conjugated phalloidin (Molecular Probes, USA) for 120 min. After washing with PBS, cells were incubated in DAPI for 10 min at a dilution of 1:1000. All fluorescent staining was visualized using a Leica TCS NT confocal microscope with the ×63 oil immersion objective lens.

### Statistical Analysis

Statistical analyses were performed using the Student's unpaired *t*-test. Each experiment was conducted at least twice. The data presented represent means ± standard deviation (SD) of independent replicates (n = 3). Results were considered statistically significant when P ≤ 0.05.

## Results

### Proliferation effect of rBMSCs under different gravity

**Figure 1:**
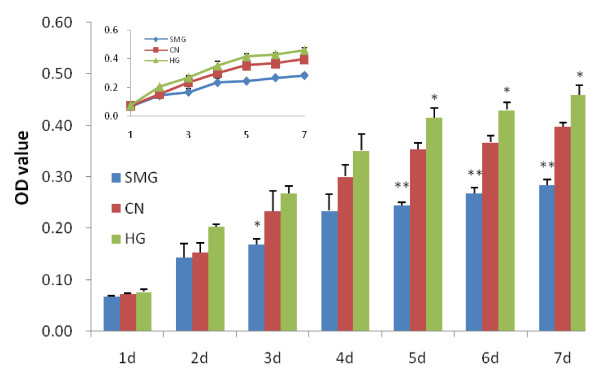
**Effects of HG/SMG on the proliferation of BMSCs**. BMSCs were plated onto glass coverslips, transferred to a biocompatible polyethylene culture bag [27], then cultured under HG or SMG conditions for 1--7 days. The cells were fixed with 4% paraformaldehyde and proliferation was assessed by the methylene blue method. HG promoted the proliferation of rBMSCs and SMG inhibited proliferation. * SMG or HG versus Control (CN), p < 0.05, ** P < 0.01, n = 3.

We have previously demonstrated that SMG decreased the proliferation of rBMSCs during culture for 1-3 days [27]. In the present study, rBMSCs were cultured under HG/SMG conditions for 1-7 days and proliferation was assessed using the methylene blue assay. In agreement with our previous results, SMG presents significant proliferation inhibition at day 3 and rBMSCs almost stop growing after day 4. By contrast, HG promotes the growth of rBMSCs, which show a marked increase at day 5 (Fig. 1).

### Osteoblast differentiation of rBMSCs under different gravity conditions

**Figure 2:**
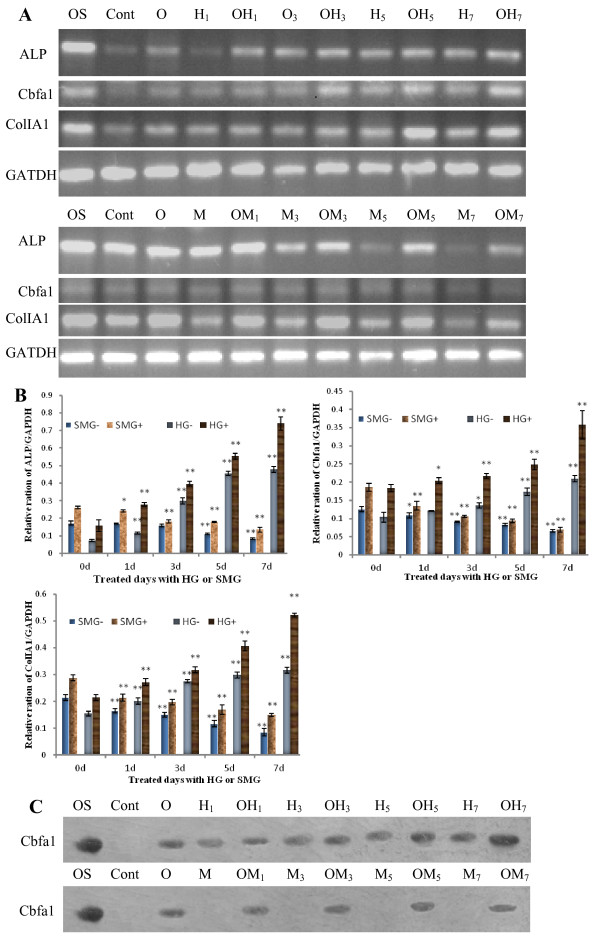
**Effects of HG/SMG on the osteogenic differentiation of rBMSCs**. rBMSCs were cultured under HG/SMG conditions with or without osteoblastic inducement medium for 1, 3, 5 or 7 days during a culture period of 14 days. RT-PCR analysis for ALP, cbfa1 and ColIA1 mRNA expressions:(A) Electrophoresis graph of PCR products, B) Gray intensity analysis of electrophoresis bands. (C) Western-blot analysis of Cbfa1 expression. OS indicates a positive control (RNA extracted from ROS17/2.8 cells); *Cont*, cells were cultured under normal conditions without inducer; O, rBMSCs were added with inducer for osteoblast; H, HG culture; M, SMG culture; numbers 1, 3, 5, 7 indicate the days of HG/SMG culture. Take samples: OH3, cells were cultured under HG for 3 day with osteogenic inducer; M7, cells were cultured under SMG for 7 days without osteogenic inducer. SMG-/HG-, SMG/HG treated without inducer; SMG+/HG+, SMG/HG treated with inducer. * p < 0.05 and ** P < 0.01, compared with relative 0 d group (Cont, O) using student *t-test *analysis(n = 3). The experiments were conducted twice. The GAPDH house-keeping gene was used as a control. HG increased the expression of ALP, Cbfa1 and ColIA1 in rBMSCs, whereas SMG decreased the expression levels.

It has been well described that microgravity inhibits osteogenesis of MSCs or osteoprogenitor cells [27-29]. In the present study, rBMSCs were cultured using a cell centrifuger under 2 G or a clinostat, to generate SMG conditions, for 1, 3, 5 or 7 days with or without inducer reagent. At day 14, all cells were treated with Trizol or protein lysis buffer, and analyzed by semi-quantitative RT-PCR or Western-blot analyses to detect the expression of osteoblastic genes such as cbfa1, ALP and ColIa1. Compared with the normal gravity group, the mRNA expression of ALP, Cbfa1 and ColIa1 increased from day 1 to day 7 in a time-dependent pattern in both the inducer treated group and the untreated group under HG conditions. The results under SMG conditions were in contrast to those seen under HG conditions, as shown in Fig. 2A, B. Cbfa1 is an essential transcript factor and a commitment factor for osteoblast differentiation [35]. A similar result was seen with Western-blot analysis (Fig. 2C) of Cbfa1 protein. These results indicate that HG increased the osteogenesis of rBMSCs and SMG inhibits its osteogenesis.

### Cardiomyogenic differentiation of rBMSCs under different gravity

**Figure 3:**
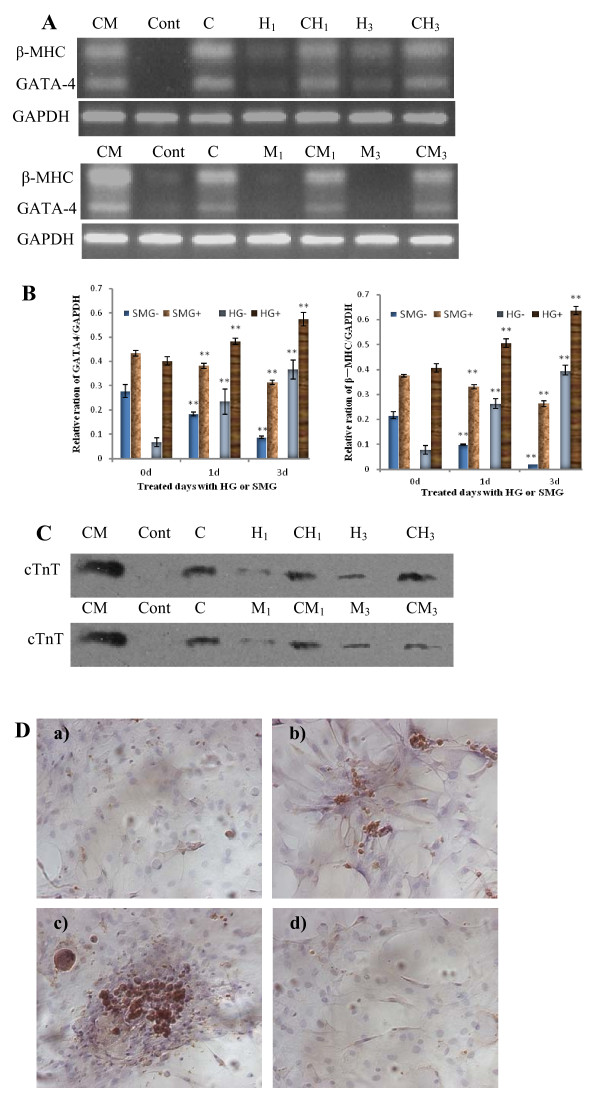
**Effects of HG/SMG on cardiomyogenic differentiation of rBMSCs**. rBMSCs were cultured under HG/SMG conditions with or without cardiomyocyte inducement medium for 1 or 3 days. RT-PCR analysis of β-MHC and GATA-4 mRNA expressions; (A) Electrophoresis graph of PCR products, (B) Gray intensity analysis of electrophoresis bands. (C) Western-blot detection of cTnT; CM, positive control, the RNA and protein were extracted from primary cardiomyocytes. *Cont*, cells were cultured under normal condition without inducer; C, rBMSCs were cultured with inducer for cardiomyocyte differentiation; H, HG culture; M, SMG culture; numbers 1, 3 indicate days of HG/SMG culture. Take samples: CH3, cells were cultured under HG conditions for 3 days with cardiomyogenic inducer; M3, the cells were cultured under SMG conditions for 3 days without inducer. SMG-/HG-, SMG/HG treated without inducer; SMG+/HG+, SMG/HG treated with inducer. * noted p < 0.05 and ** P < 0.01 compared with relative 0 d group (Cont, C) using student *t-test *analysis (n = 3). The experiments were conducted twice. The GAPDH house-keeping gene was used as a control. (D) immunocytochemistry analysis of cTnT in the cardiomyogenic differentiation of rBMSCs under HG/SMG conditions. a) The rBMSC group was used as a negative control. b) The rBMSCs treated with 5-aza only. c) The CH_3 _group strongly expressed cTnT. d) The CM_3 _group had few cTnT positive cells. Magnification × 200. HG increased the expression levels of GATA-4, β-MHC and cTnT in rBMSCs, whereas SMG decreased the expression levels.

It has been well documented that BMSCs can differentiate into cardiomyocyte with 5-aza. rBMSCs were cultured under HG/SMG conditions for 1 or 3 days respectively; then further cultured for a total 21 days in normal culture condition. The expression levels of the cardiomyocyte-specific markers cTnT, β-MHC, GATA4 were analyzed by RT-PCR or Western-blot assay. In the non-inducement group, BMSCs showed almost no expression of β-MHC and GATA4 under normal gravity and SMG conditions, but low levels under HG conditions, and expression levels in a time-dependent manner. This indicates that HG can slightly increase BMSC differentiation into cardiomyocytes without chemical inducement. In the inducement group, GATA4 and β-MHC were expressed at high levels. The expression levels of GATA4 and β-MHC in the HG group were increased compared with the control group. The BMSCs cultured under SMG conditions with cardiomyogenic inducer for 3 days (CM_3 _group) expressed lower levels of GATA4 and β-MHC compared with the control group (Fig. 3A, B).

To further determine the effects of HG and SMG on the cardiomyogenic differentiation of rBMSCs, we assessed levels of cTnT protein by immunocytochemistry and Western-blot analyses. Brown deposition indicates cellular expression of cTnT. The changes in cTnT protein expression (Fig. 3C) were markedly similar to changes in the expression levels of GATA4 and β-MHC. HG increased the expression level of cTnT, whereas SMG decreased the expression level, compared with the control group. Cells treated with 5-aza and HG for 3 days showed much stronger brown deposition than control cells and cells that were only treated with 5-aza. The CM_3 _group had few cTnT positive cells (Fig. 3D) compared with normal and HG groups. The cTnT-positive cells were spindle-shaped or column-shaped, had a fibroblast-like morphology and were connected with adjoining cells. These results indicate that the cells that strongly expressed cTnT had cardiomyocyte characteristics (Fig. 3D).

Taken together, analysis of the expression of the cardiomyocyte-specific molecular markers cTnT, GATA4 and β-MHC indicated that HG promoted cardiomyogenic differentiation of rBMSCs whereas SMG inhibited this process.

### Adipogenic differentiation of rBMSCs under different gravity

**Figure 4:**
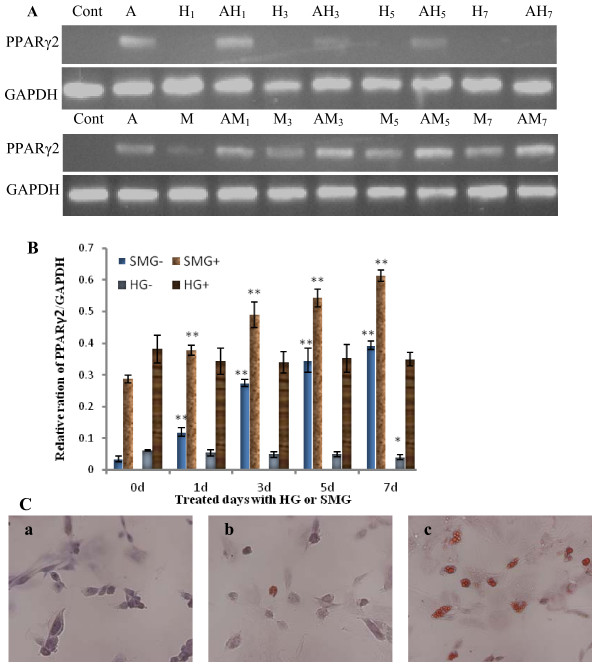
**Effects of HG/SMG on the adipogenic differentiation of rBMSCs**. rBMSCs were cultured under HG/SMG conditions with or without adipogenic inducement medium for 1, 3, 5 or 7 days of a total culture period of 14 days. RT-PCR analysis of PPARγ2 mRNA expression. (A) Electrophoresis graph of PCR products, (B) Gray intensity analysis of electrophoresis bands. *Cont*, cells were cultured under normal condition without inducer; A, rBMSCs were cultured with inducer for adipocyte differentiation; H, HG culture; M, SMG culture; the numbers 1, 3, 5, 7 indicate the days of HG/SMG culture. Take samples: AH7, cells were cultured under HG conditions for 7 days with adipogenic inducer; M3, cells were cultured under SMG conditions for 3 days without inducer. SMG-/HG-, SMG/HG treated without inducer; SMG+/HG+, SMG/HG treated with inducer. * noted p < 0.05 and ** P < 0.01 compared with relative 0 d group (Cont, A) using student *t-test *analysis (n = 3). The experiments were conducted twice. The GAPDH house-keeping gene was used as a control. (C) Oil red-O staining to detect the adipogenic differentiation of rBMSCs under HG and SMG conditions. a) The control group. b) The AH_7 _showed few oil droplets. c) The AM_7 _group contained oil droplets in the cells. Magnification × 200. SMG conditions increased the expression of PPARγ2.

As the above results show, HG and SMG conditions affected the differentiation of BMSCs into cardiomyocytes and osteoblasts, both of which are force-sensitive cells *in vivo*. It is unknown how the different gravity conditions affect adipocytes, a force-insensitive cell. BMSCs were cultured using a centrifuger to get 2 G HG or a clinostat to simulate microgravity conditions for 1 day, 3 days, 5 days and 7 days with or without inducer reagent. At day 14, cells were lyzed with Trizol for total RNA extraction or fixed with paraformaldehyde for oil red staining. The expression of the adipocyte-specific marker PPARγ2 was analyzed by semi-quantitative RT-PCR. In the non-inducement group, BMSCs expressed almost undetectable levels of PPARγ2 when cultured under normal gravity and HG conditions, but expressed increased levels in a time-dependent manner when cultured under SMG conditions. In the inducement group, HG reduced the expression of PPARγ2 and simulated microgravity increased its expression in a time-dependent pattern. After 7 days, more SMG-cultured cells were positive for oil red staining compared with the HG and static control groups. These results indicate that SMG conditions promote rBMSC differentiation into adipocytes, which are force-insensitive cells (Fig. 4).

### The effects of HG on differentiation into force-sensitive cells

**Figure 5:**
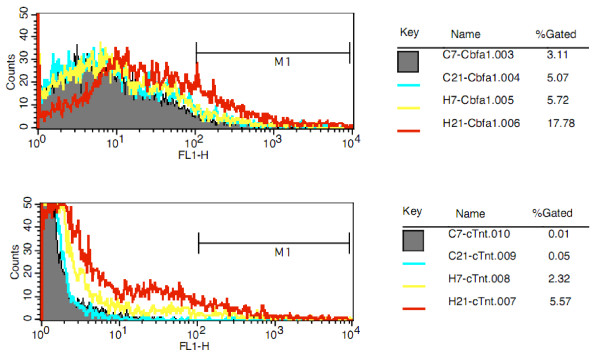
**Effects of HG on rBMSCs differentiation into force-sensitive cells**. rBMSCs were cultured under HG conditions for 7 or 21 days. Cells were then analyzed by FACS with a cTnT-specific monoclonal antibody to detect cardiomyogenic differentiation, or a Cbfa1-specific antibody to detect osteoblastic differentiation. C, static control group; H, HG group. HG increased the numbers of Cbfa1- and cTnT-positive cells compared with the control group, particularly after HG culture for 21 days.

To confirm above results, we further analyzed the effects of HG conditions on the differentiation of BMSCs into force-sensitive cells. Uninduced BMSCs were cultured under HG for 7 days and 21 days, then analyzed by FACS analysis with monoclonal antibodies for Cbfa1 and cTnT. As shown in Fig. 5, HG conditions increased the levels of Cbfa1- or cTnT-positive cells compared with the static control group, particularly at 21 days. After culture under HG conditions, the number of Cbfa1-positive cells increases presenting a time-dependent manner, which indicates that HG can promote this process. Also, HG slightly increased the differentiation of BMSCs into cardiomyocytes. In the static control group, there was no difference in the number of cTnT-positive cells between 7 and 21 days, but in the HG treatment group the number of positive cells increased, particularly at 21 days. This result was in agreement with RT-PCR mRNA analysis results (Fig. 3A, B).

### Effects of different gravity conditions on the cytoskeleton of rBMSCs

**Figure 6:**
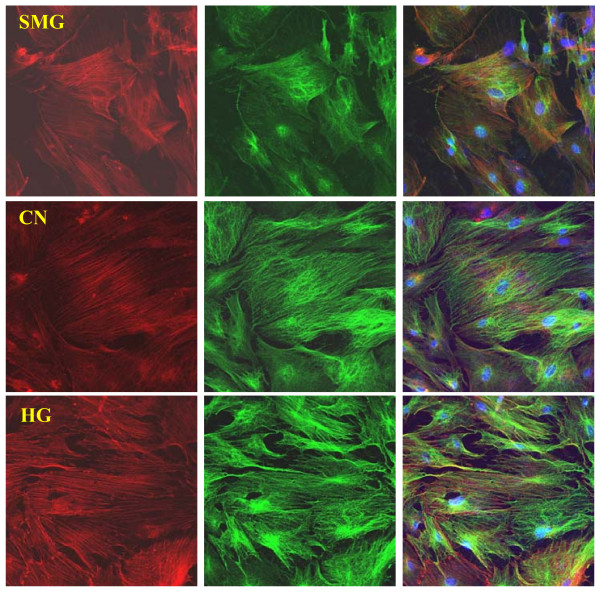
**Effects of HG/SMG on the cytoskeleton of BMSCs**. rBMSCs were cultured under HG/SMG conditions for 7 days, then fixed with 4% paraformaldehyde and stained for microfilaments with Texas red isothiocyanate-conjugated phalloidin (red), microtube cytoskeleton with FITC--conjugated antibody (green) and nucleolus with DAPI (blue). In the SMG group, microfilaments appeared thinner and abnormally distributed, and microtubules appeared diffuse, compared with the control group (CN). In the HG group, the diameters of microfilaments and microtubules appeared to increase. Magnification ×63 oil immersion objective.

The cytoskeleton is important for the regulation of differentiation and is sensitive to gravity changes [36,37]. Compared with normal gravity conditions, the distribution and structure of microfilaments and microtubules were clearly rearranged under HG/SMG conditions. Under SMG conditions, microfilaments appeared thinner and abnormally distributed, and microtubules appeared diffuses; whereas HG conditions increased the polymerization of microfilaments and microtubules, which appeared thicker and stronger (Fig. 6).

### Effects of different gravity on ERK1/2 phosphorylation of rBMSCs

**Figure 7:**
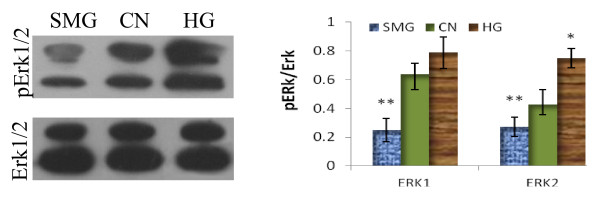
**Effects of HG/SMG on the activity of ERK1/2**. rBMSCs were cultured under HG/SMG conditions for 7 days, then analyzed by Western blotting for Erk1/2 and phospho-Erk1/2. There was no difference in the expression level of ERK among the three groups, but the phosphorylation level of ERK1/2 strongly increased in the HG group and decreased in the SMG group. The right image shows the gray analysis of the Western-blot bands. * p < 0.05 and ** p < 0.01, compared with relative CN group (n = 3).

MAPK-Erk signaling is involved in osteogenetic and cardiomyogenenic differentiation regulation, and is also an important pathway in BMSC proliferation and activity. The ERK1/2 phosphorylation of rBMSCs was detected by Western-blot analysis after HG/SMG culture without inducement for 3 days. Although there was no clear change in the ERK protein level with HG/SMG conditions, the phosphorylation of ERK1/2 was strongly increased in the HG group and decreased in the SMG group. The ratio of phospho-Erk to Erk was down-regulated by SMG and up-regulated by HG conditions (Fig. 7).

## Discussion

Adult stem cells persist throughout life and are involved in repair or replacement of cells in certain tissues in response to traumatic events or natural cell turnover, which are important to maintain body homeostasis. Adult stem cells have great potential for tissue engineering and regenerative or degenerative medicine therapies [3,38]; but some problems remain to be resolved, such as increasing the committed differentiation efficiency and providing sufficient seed cells and delivery methods. As an important progenitor cell, BMSCs can differentiate into multiple cell types and can migrate to injured areas [39]. During the past 40 years, human experience of spaceflight has shown that exposure to microgravity affects almost all human physiological systems. Previous studies, both our own and those of other researchers, have demonstrated that microgravity inhibits the proliferation and osteogenesis of BMSCs and increases the rate of adipogenesis [27,28]. It is not yet known if altered differentiation of BMSCs contributes to spaceflight-induced physiological changes, but offers an interesting explanation and provides a chance to interpret the function of gravity during life and evolution. In the present study, we further demonstrated that different gravity conditions (SMG or HG) have important roles in guiding the differentiation of BMSCs.

In the present study, a cell centrifuger was used to generate HG conditions and a clinostat was used to generate SMG conditions [40-43], both of which have been widely used to investigate the effects of different gravity conditions on tissue and cell functions during ground-based investigations [27,44]. The cell vessels in the clinostat were horizontally rotated and those in the centrifuger were vertically rotated. The vessels were fully filled with medium, so that the culture medium and individual cells rotate synchronously with the vessel and result in extremely low shear stress and cell turbulence. By adjusting the rotating speed, cells can gain different gravity values in the centrifuger.

An important finding using the centrifugation and clinorotation methods in this study is the different effects on the differentiation of rBMSCs into force-sensitive or force-insensitive cells. It has previously been demonstrated that SMG inhibited osteogenesis and promoted adipogenesis under induction condition [28]. In the present study, we further demonstrated that SMG inhibited differentiation into cardiomyocytes and osteoblasts with or without inducer. Osteoblasts and cardiomyocytes are both force-sensitive cells *in vivo*, and their functions are related to mechanical stress. During spaceflight, microgravity results in disuse bone loss, muscle and heart atrophy, but increases adipogensis in marrow and other tissues [21-29]. After mature development, bone, muscle and heart tissues require reconstruction to cope with the demands stresses such as weight bearing and mechanical force. As a type of mechanical force, gravity is important for the differentiation of adult stem cells. On the other hand, HG promoted BMSC differentiation into cardiomyocytes and osteoblasts, but decreased the differentiation into adipocytes. Centrifuge-induced artificial gravity conditions have been used to prevent cardiovascular deconditioning and bone loss induced by microgravity exposure [45]. HG affects the mRNA expression in different rat tissues [46]. In the present study, we found that HG increased the expression of cardiomyocyte markers (cTnT, GATA4 and β-MHC) and osteoblast markers (ALP, Cbfa1 and ColIA1) in BMSCs cultured under induction conditions, but decreased the expression of the adipocyte-specific transcript factor PPARγ. In addition, the number of Cbfa1- or cTnT-positive cells was increased after HG culture without inducer, which further demonstrated that HG conditions can promote BMSC differentiation into force-sensitive cells and inhibit differentiation into force-insensitive cells. This finding has particular potential for regenerative medicine and developmental biology. HG and inducer conditions can promote BMSC committed differentiation, and SMG conditions may provide an environment to successfully expand stem cell populations *in vitro *without requiring culture supplements that can adversely affect stem cell-based transplantations [47]. It should be noted that injection of the centrifuged MSCs into the area of a rat heart affected by myocardial infarction can markedly improve the biological and functional benefits of the heart (manuscript from Shukuan Ling).

We also investigated the effects of HG/SMG on rBMSC activity and proliferation. As reported previously, SMG inhibited rBMSC proliferation during 1-3 days culture [27]. In the present study, we demonstrated that proliferation continues to decrease with longer culture (1-7 days) and that HG promotes rBMSC proliferation. There was an increase during 1-4 days, but the difference was not significant compared with normal condition. The increase became more significant during days 5-7. However, microgravity had marked inhibitory effects at the third day. The HG G value (only 2 G) might be too low to significantly increase the proliferation rate during the first 4 days, but there might be an accumulated effect by day 5. This is consistent with previous results [48,49] and those of a previous study investigating the changes in bone marrow hematopoietic stem cells (HSC) under the HG/SMG conditions [26]. Kostenuik [50] demonstrated that hindlimb suspension for 5 days significantly decreased the proliferation of BMSCs. Takemura [51] reported that HG increases the activity of DNA polymerase, which may lead to increased cell proliferation.

It is known that extracellular signal-regulated protein kinases (ERKs) of the mitogen-activated protein (MAP) kinase pathway signaling are involved in the proliferation and differentiation processes of osteoblast and cardiomyocyte differentiation [52,53]. One potential mechanism for reduced osteoblastic differentiation by SMG involves disruption of type I collagen (Col I)-integrin interactions and reduced integrin signaling [42,54]. The phosphorylation level of ERK1/2 was assessed to understand its potential mechanism. As would be expected, the phosphorylation level of ERK1/2 was increased with HG and decreased with SMG. The cytoskeleton has been shown to be affected by the extracellular microenvironment and can transduce mechanical stress signals [55,56]. Destroyed microfilaments may block cells in G0/G1 phase of the cell cycle and decrease proliferation. As previously reported, SMG led to collapse of microfilaments and the microtube cytoskeleton [27]. In the present study, we provide the first evidence that HG increases organization of microfilament and microtubules, which may increase cell activity. Overall, gravity factors affect the activity and differentiation of BMSCs, which may involve the cytoskeleton and ERK signal transduction pathway. Further study is required to determine the progress of signal transduction.

## Conclusion

In conclusion, we show that HG promotes rBMSC differentiate into force-sensitive cells, cardiomyocytes and osteoblasts, whereas SMG promotes differentiation into force-insensitive cells, namely adipocytes. Gravity is an important factor that affects the differentiation of rBMSCs. This finding has significant potential for regenerative medicine, tissue engineering and stem cell-based therapy. Furthermore, these results also demonstrate the use of effective measures to investigate the potential mechanisms and clinic applications of stem cell differentiation.

## Competing interests

The authors declare that they have no competing interests.

## Authors' contributions

ZQD, YHL and YMW participated in the design of this study. SKL carried out the flow cytometric assay experiment and data analysis. YH and ZQD performed all the other experiments and HYZ assisted them. YH and ZQD prepared the manuscript and YHL reviewed it.
